# Lean Six Sigma Methodology in the Implementation of a Standardized Health Literacy Assessment in a Safety Net Internal Medicine Residency Clinic

**DOI:** 10.3928/24748307-20181217-01

**Published:** 2019-02-05

**Authors:** Rose Penix, James Kish, Matthew Gustovich, Michelle Cudnik

## Abstract

**Background:**

Lean Six Sigma is a set of techniques intended to improve processes by greatly reducing the probability that errors will occur. The Lean Six Sigma approach was used to engage staff and clinicians in standardizing the assessment of patient health literacy in an academic safety net clinic.

**Brief description of activity:**

Clinicians were surveyed, and a chart review was conducted to attain baseline information regarding the uniformity of health literacy assessment and documentation in the clinic. A workgroup used the survey data and Lean Six Sigma quality improvement tools to determine the root cause, which was the lack of standardization.

**Implementation:**

To address this root cause, a standardized process for health literacy assessment in the clinic was developed; an assessment tool was selected, support staff were trained, and the new process was initiated. A support staff focus group was conducted, and a second clinician survey was disseminated to gauge perception of the new health literacy assessment and documentation processes. Quantitative data were collected to assess the coverage achieved by the new processes. Monthly reporting and staff feedback sessions were conducted to ensure continued efficacy of the protocol.

**Results:**

Standardized assessment and documentation processes were effectively implemented using the Lean Six Sigma approach in an academic safety net clinic. Providers and rooming staff were satisfied with the new processes. Rooming staff input and buy-in were critical to successful implementation.

**Lessons learned:**

Our findings suggest that Lean Six Sigma tools and methodologies can be appropriate for health care quality improvement and process standardization in an academic safety net clinical setting. **[*HLRP: Health Literacy Research and Practice*. 2019;3(1):e25–e30.]**

**Plain Language Summary:**

A growing body of evidence supports the role of Lean Six Sigma (LSS) tools and processes for the assessment, design, and implementation of the quality improvement in the health care industry. This study explored the use of LSS on the standardization and documentation of health literacy assessment of patients in an academic safety net clinic. Findings suggest that LSS is an appropriate method of standardizing assessment and documentation of health literacy in this clinical setting.

Health literacy is the degree to which people have the capacity to obtain, process, and understand basic health information and services needed to make appropriate health decisions (Sørensen et al., 2012), and it is directly correlated with health outcomes (DeWalt et al., 2004).

The Internal Medicine Center is an academic urban outpatient facility that averages 16,000 adult patient appointments per year (**Table 1**). The clinic is part of an Accountable Care Organization and is a Level 3-Certified “Patient Centered Medical Home” (PCMH), meaning that the health care organization is structured to coordinate patient care through a variety of clinicians across the continuum of care (Longworth, 2011).

The National Committee for Quality Assurance (NCQA) is a top health care accreditation organization. To achieve accreditation as a PCMH, organizations must demonstrate that they have met NCQA standards and guidelines, which includes the assessment of patient health literacy. Through research of the staff chart review for the Internal Medicine Center accreditation application, it was discovered that clinicians were not assessing patient health literacy in a standard fashion. This was brought to the attention of faculty clinicians during a monthly faculty meeting, and the faculty decided that improving health literacy assessment (HLA) and documentation was a priority. Faculty clinicians, resident clinicians, rooming staff, and research staff volunteers formed a health literacy workgroup with two Process Engineers from the Quality Improvement Department to guide the group effort. The Process Engineers had achieved Lean Six Sigma Green Belt Certification. Because this is a Quality Improvement initiative, no institutional review board approval was needed for this initiative.

Lean Six Sigma methods and tools are the standard system-wide approach to process improvement and were selected to address these issues (Orr & Powers, 2016).

Lean Six Sigma is a performance improvement methodology that incorporates effectiveness to reduce variation in an existing process. The health literacy workgroup aimed to develop and test methods to standardize HLA and identify patients with low health literacy. The focus of this report is to describe Lean Six Sigma methods that may be used to implement HLA in similar settings.

## Using the Five Stages of Lean Six Sigma to Standardize Health Literacy Assessments

### Define

The project was organized, and a project team was assembled. The customer and business objectives (goals) were defined and validated by all “customers” of the process. The customers in this case were the clinicians and clinical staff, specifically medical assistants and nurses responsible for rooming patients in the clinic. This phase began in December 2016.

All clinicians were surveyed about HLA use (**Table A**) via email and about one-half of those responded. Responses indicated that 8% of clinicians were assessing patient health literacy. From 1 month's completed office visits, a sample of patient charts were selected at random and a review was conducted by the health literacy work-group research staff. The chart review found that only 3 of 50 (8%) clinicians documented an HLA and that a variety of tools designed by the clinicians themselves were being used. The health literacy workgroup determined that the electronic medical record's (EMR) built-in HLA tool was not a validated instrument and that it was too time consuming to be practical in a clinical setting. Also, due to an internal technical issue, not all clinicians had access to this tool in the EMR. The group decided to select a different tool.

The SIPOC (suppliers, inputs, process, outputs, customers; **Figure A**), a visual tool for documenting business process from beginning to end, was used by the workgroup. Although the existing process involved clinicians conducting the HLA, the group determined that it would be more appropriate for rooming staff to be responsible for patient HLA.

A tool called the voice of the customer (VOC; **Table B**) is intended to clarify what the customers of the process value and then translate the information into quantifiable metrics. The VOC included the clinician survey data and rooming staff input collected at workgroup meetings. During the development of the VOC table, the workgroup concluded that the HLA was valuable to clinicians, should not offend patients, and should not be an imposition to rooming staff work flow. Goals for the project based on the VOC were (1) complete HLAs for 80% of new patients, (2) ensure the HLA took less than 5 minutes to complete, and (3) develop an accessible standardized process for documentation of results.

### Measure

Baseline (current state) data were collected to validate the problem's existence. The aforementioned survey indicated that a small percentage, approximately 8%, of clinicians stated that they were consistently conducting HLAs. To further validate the problem's existence, two additional research staff used the same measurement system and sampling approach as the earlier chart review. They verified baseline findings that only 3 of 50 (6%) charts contained documentation of an HLA and that the HLA tools and documentation were inconsistent. The measure phase was completed in February 2017.

### Analyze

In February 2017 the workgroup invited clinicians from the Family Medicine Center, a similar residency clinic to discuss their experiences with their prior temporary HLA implementation. The Family Medicine Center clinic is located on the same campus as the Internal Medicine Center clinic but serves children as well as adults. Data and process information were examined during a brainstorming session to determine the critical root causes of the problem, which included a lack of access to a standardized tool and a lack of accessibility of patient HL scores. Root causes were validated through observations of rooming procedure by health literacy workgroup members and became the basis for which solutions were developed.

### Improve

Solutions were implemented to specifically address the critical root causes. Potential risks included the possibility that patients would react poorly to the HLA questions. Rooming staff felt that patients would be receptive but were empowered to skip the HLA if it seemed that administration would negatively affect patient satisfaction.

Another potential risk included rooming staff neglecting to perform an HLA due to time constraints. To address the root causes, the workgroup (including rooming staff) selected a validated HLA instrument called the Brief Health Literacy Assessment (BRIEF) (Louis, Arora, & Press, 2014). The BRIEF takes less than 2 minutes to administer verbally. A fillable version of the BRIEF was programmed into the clinic's EMR. When rooming a patient, rooming staff could verbally administer and document patients' responses along with the total score. There is a total of 25 points available, and 9 or fewer points indicated low health literacy (**Table C**). Clinical staff and physicians were given an educational manual about administering the BRIEF, which included information such as standardizing methods of questioning patients, how to respond if the patient declined, and how to score the assessment. Rooming staff were trained in interactive group sessions by research staff on the use of this tool.

A 2-month long pilot commenced in March 2017 to validate that the solution had the desired effect on the process. The pilot phase focused on assessing and documenting HLA scores for patients “new to the practice.” During the pilot, the rooming staff (three medical assistants and two nurses) conducted HLAs while rooming the patient and would alert physicians of the low assessment results by entering low health literacy scores in the “chief complaint” of the patient's chart. Physicians would then document low health literacy in the patient chart by adding an *International Statistical Classification of Diseases and Related Health Problems*, 10th revision code (World Health Organization, 1992) for low literacy to the patient problem list.

Two weeks into the pilot, a focus group moderated by research staff was conducted to gauge perceptions of the HLA process and tool among the rooming staff (nurses and medical assistants). Thematic analysis was used to analyze responses. Nurses and medical assistants (the clinic's rooming staff) perceived the process and the administration of the patient HLA positively. The group's opinion was that patients also received the assessment favorably.

Three weeks into the pilot phase, a second email survey was disseminated to clinicians (**Table D**). About 40% of clinicians responded, and 64% of those that had seen patients indicated that their awareness of the patient's health literacy score altered the way in which they interacted with the patient.

Internal Medicine Center's Information Technology and Analytics Reporting division constructed a report that tracked HLA smart phrase use in the clinic. This report is provided monthly to the health literacy workgroup research staff for evaluation. In month 1 of the pilot, 85% of patients were properly assessed, and 81% of the patients had the score documented properly. In month 2 of data collection, 91% of patients were properly assessed, and 63% of the patients had the score documented properly. Approximately 24% of assessed Internal Medicine Center patients were found to have low health literacy. The HLA completion and documentation rates exceeded project goals, implying that the pilot process properly addressed the critical root causes, and the health literacy workgroup determined to continue with this process.

A training plan was developed to address each functional group: rooming staff, residents, and faculty. The plan included “new knowledge and skill requirement,” “estimated length/format,” “owner,” “start date,” and “completion date.” Once all functional groups received training, the process was implemented permanently.

### Control

Along with the roll out, controls were put into place to ensure the new process is maintained and to protect against backsliding. Health literacy workgroup research staff receives monthly reports documenting completed HLA surveys for the practice. The new HLA process will be revisited via faculty meetings if less than 80% of appropriate patients are being screened. The control phase was implemented immediately with the roll out of the pilot and is ongoing.

## Discussion and Implications for Practice

The Lean Six Sigma data-driven, process-improvement methodology provides meaningful results and customer satisfaction. This project aimed to explore methods to overcome barriers to the implementation of a standardized HLA practice. Clinician and clinical staff engagement throughout the planning and pilot project was a key factor in successfully implementing an HLA protocol. It is important to involve the customers of the process and also those who work most closely within the process to ensure sustainability. The Internal Medicine Center's HLA process and HLA documentation process was designed using qualitative analysis, survey, and focus group assessments. Lean Six Sigma methodology was instrumental in the successful roll out, assessment of, and sustainability of this project.

Although the implications for health literacy screening scores are dependent on how they are used in the clinical setting, the use of Six Sigma methodology and the inclusion of “customers” in the design and implementation of a standardized HLA process and a standardized HLA documentation process were found by this assessment to be valuable. Further research should be conducted on the use of Lean Six Sigma tools in standardizing processes associated with patient HLA and health literacy documentation.

## Figures and Tables

**Table 1:** Clinical Staff at the Internal Medicine Center

**Title**	**Role**	**Number of Each**
Resident physicians	Clinicians	62
Pharmacy students	Learners	16
Faculty physicians	Clinicians	11
Office personnel	Support staff	9
Nurses	Rooming staff	8
Pharmacy residents	Learners	4
Medical assistants	Rooming staff	4
Behavioral health consultants	Staff	2
Pharmacist	Clinicians	1
Part-time psychiatrist	Staff	1

**Figure A.**
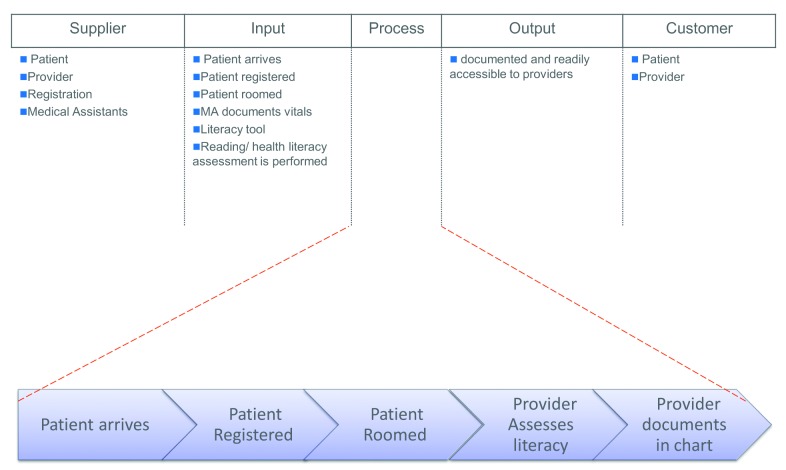
Schematic of SIPOC (supplier, input, process, output, customer) analysis.

**Table A:** Questions in the Initial Clinician Survey

Do you perform a health literacy assessment? Do you assess all of your patients? Do you use a validated instrument? Where do you document the results?

**Table B:** Voice of the Customer Document

	**Customer**	**Voice of the Customer**	**Critical To Quality**
Clinicians		See HL as valuable to patient care Believe that support staff would be appropriate HLA conductors, as it would reduce physician in-room time	80% of patients have HL assessed and documented in standardized place
Clinicians		All IMC patients complete an HLA	80% of appropriate patient visit types are assessed (not sick visits)
Clinicians		Would like HL information to become available to the clinician with minimal effect on physician in-room time	HLA takes <5 minutes to complete HLA completed outside of physician in-room time
Rooming staff		Believes that HL is valuable and that there is adequate time during rooming to conduct HLA	HLA takes <5 minutes to complete

Note. HL = health literacy; HLA = health literacy assessment; IMC = Internal Medicine Center.

**Table C:** Brief Health Literacy Assessment Tool

How often do you have someone help you read hospital materials? 1. *Always*, 2. *Often*, 3. *Sometimes*, 4. *Occasionally*, 5. *Never* How often do you have problems learning about your medical condition because of difficulty understanding written information? 1. *Always*, 2. *Often*, 3. *Sometimes*, 4. *Occasionally*, 5. *Never* How often do you have a problem understanding what is told to you about your medical condition? 1. *Always*, 2. *Often*, 3. *Sometimes*, 4. *Occasionally*, 5. *Never* How confident are you filling out medical forms by yourself? 1. *Not at all*, 2. *A little bit*, 3. *Somewhat*, 4. *Quite a bit*, 5. *Extremely*

**Table D:** Postclinician Survey

Did the health literacy score change the way you interacted with your patients? Yes/No Is there some way that we can make the process easier or more accessible to you?
